# Evaluation of novel highly specific antibodies to cancer testis antigen Centrin‐1 for radioimmunoimaging and radioimmunotherapy of pancreatic cancer

**DOI:** 10.1002/cam4.2379

**Published:** 2019-07-16

**Authors:** Rubin Jiao, Kevin J. H. Allen, Mackenzie E. Malo, Muath Helal, Zewei Jiang, Karishma Smart, Susan V. Buhl, David Rickles, Ruth A. Bryan, Ekaterina Dadachova

**Affiliations:** ^1^ College of Pharmacy and Nutrition University of Saskatchewan Saskatoon Saskatchewan Canada; ^2^ Albert Einstein College of Medicine Bronx New York; ^3^ RadImmune Therapeutics Tarrytown New York

**Keywords:** ^177^Lutetium, ^213^Bismuth, centrin1, pancreatic adenocarcinoma, radioimmunotherapy, SPECT/CT imaging

## Abstract

**Background:**

Pancreatic ductal adenocarcinoma (PDAC) accounts for >90% of pancreatic malignancies, and has median survival of <6 months. There is an urgent need for diagnostic and therapeutic options for PDAC. Centrin1 (CETN1) is a novel member of Cancer/Testis Antigens, with a 25‐fold increase of *CETN1* gene expression in PDX from PDAC patients. The absence of selective anti‐CETN1 antibodies is hampering CETN1 use for diagnosis and therapy. Here we report the generation of highly specific for CETN1 antibodies and their evaluation for radioimmunoimaging and radioimmunotherapy (RIT) of experimental PDAC.

**Methods:**

The antibodies to CETN1 were generated via mice immunization with immunogenic peptide distinguishing CETN1 from CETN2. Patient tumor microarrays were used to evaluate the binding of the immune serum to PDAC versus normal pancreas. The antibodies were tested for their preferential binding to CETN1 over CETN2 by ELISA. Mice bearing PDAC MiaPaCa2 xenografts were imaged with microSPECT/CT and treated with ^213^Bi‐ and ^177^Lu‐labeled antibodies to CETN1.

**Results:**

Immune serum bind to 50% PDAC cases on patient tumor microarrays with no specific binding to normal pancreas. Antibodies demonstrated preferential binding to CETN1 versus CETN2. Antibody 69‐11 localized to PDAC xenografts in mice in vivo and ex vivo. RIT of PDAC xenografts with ^213^Bi‐labeled antibodies was effective, safe, and CETN1‐specific.

**Conclusions:**

The results demonstrate the ability of these novel antibodies to detect CETN1 both in vitro and in vivo; as well, the RIT treatment of experimental PDAC when radiolabeled with ^213^Bi is highly efficient and safe. Further evaluation of these novel reagents for diagnosis and treatment of PDAC is warranted.

## 1 INTRODUCTION

**Figure 1:**
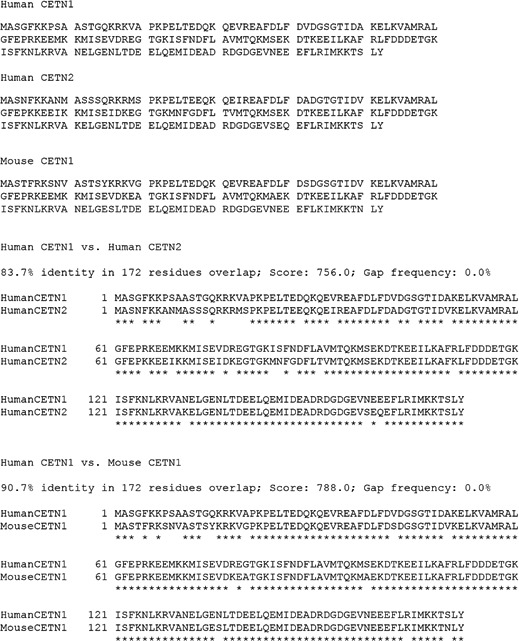
Comparative sequences of human and mouse CETN1 and CETN2

Pancreatic ductal adenocarcinoma (PDAC), which accounts for more than 90% of pancreatic malignancies, is the 3rd leading cause of cancer death in the United States with a rising incidence,^1^ and a median survival of less than 6 months. This cancer rapidly disseminates to the lymphatic system and distant organs. Due to its aggressive nature the disease is often already at an incurable stage when it is first diagnosed. Although many other solid malignancies can be biopsied, a biopsy of the pancreas is a very invasive procedure, recommended only when a mass suspected to be PDAC is causing an obstruction, or when there is evidence of metastasis and a tissue biopsy is necessary to direct chemotherapy. Accordingly, there is a strong need for the development of detection, diagnostic, and therapeutic options for PDAC. Centrin1 (CETN1) and Centrin2 (CETN2) are multi‐functional calcium‐binding phosphoproteins with four Ca^2^+‐binding domains that are present in all eukaryotes. CETN1 and CETN2 are mainly expressed in the centrosomes and the microtubules and have an essential role in mitosis and meiosis. *CETN1* is an intron‐less gene located on chromosome 18 and it is thought to have arisen from a retrotransposition using *CETN2* mRNA.^2^ CETN1 is also a compensatory protein for inactivation of X‐linked CETN2 during spermatogenesis and, in contrast with CETN2, is highly expressed during neonatal development.^2^ CETN2 is highly conserved across eukaryotes and is encoded by an X‐linked gene.^3^ It is believed to regulate DNA damage recognition during nucleotide excision repair^4^ and phosphorylated CETN2 is required for centriole separation during centrosome duplication in cell replication.^4^ Sequencing of *CETN1* and *CETN2* showed approximately 80% homology between the two proteins (Figure1).

Research indicates CETN1 is testis‐ or photoreceptor‐specific and not expressed in other organs.^5^, ^6^ It has been identified as a novel member of a growing family of proteins called Cancer/Testis Antigens (CTAs), with qPCR studies indicating a 25‐fold increase of *CETN1* expression in 50% of tumors from patients with pancreatic and prostate cancers.^2^ In addition, knockdown of CETN1 inhibits the cell proliferation in breast cancer, thus pointing to the function of CETN1 in tumor development.^7^ Most CTAs are considered hub proteins because they can interact with numerous proteins and form networks that are not normally present in cells.^2^, ^8^ Importantly, since both testes and photoreceptors are immunoprivileged site, CETN1 in this organ would not be detected by CETN1‐specific antibodies,^2^, ^9^ which should minimize toxicity to this organ. However, while CETN1 could be an ideal target for the diagnosis and treatment of PDAC and prostate cancer in patients expressing this biomarker due to its specific expression and role in cell division ; the anti‐CETN1 antibodies available on the market do not sufficiently discriminate between CETN1 and CETN2, hampering their use as effective oncology biomarker detectors and therapeutics. Here, we report the generation of highly specific for CETN1 antibodies and their evaluation in patients’ tumor microarrays and for radioimmunoimaging and radioimmunotherapy (RIT) of experimental PDAC.

## 2 MATERIALS AND METHODS

### 2.1 Immunization

Peptides were synthesized by Genemed Synthesis Inc (San Antonio, TX). Peptide sequences were selected based on a comparison of the amino acid sequences of *CETN1* and *CETN2* (Figure 1). A 15‐amino acid residue peptide comprising the sequence [KPSAASTGQKRKVAP] was chosen as the most likely to be immunogenic. This peptide was derived from the amino terminus of the CETN1 protein beginning at the seventh amino acid residue. Peptide antigen was prepared as an unmodified peptide. Peptide antigen was additionally prepared as a conjugate with poly‐L‐lysine. Several antigenic peptides can be linked to a single poly‐L‐lysine backbone, thus rendering this antigen more likely to stimulate antibody production. A cDNA clone of the HIS‐tagged human CETN1 protein was purchased from GeneCopoeia. The protein was expressed and purified by the Macromolecular Therapeutics Development Facility, Albert Einstein College of Medicine, NYC, USA.

Five BALB/c mice were immunized by injection with an emulsion of the poly‐L‐lysine peptide antigen and complete Freund's adjuvant. The mice were boosted after two weeks and six weeks with the poly‐L‐lysine conjugated peptide antigen and incomplete Freund's adjuvant. At 4 months and 6 months following the initial injection, the mice were re‐boosted with the modified peptide antigen. The mice were rested for 10 months and most promising mouse was boosted with purified CETN1‐HIS tagged protein, and sacrificed 3 days following the final boost. Sera samples were collected 2 weeks after each boost to test for binding to CETN1 by Enzyme‐linked immunosorbent assay (ELISA). Pre‐immune sera were used for negative controls.

### 2.2 Production of hybridomas

Murine B cell‐myeloma hybridomas were produced by fusing myeloma cells, Ag8.653 or NSO^bcl2^ with murine B cells. The spleen was removed from the immunized mouse, cells isolated by balloon method and the RBC lysed. Spleen cells were then washed and mixed with myeloma cells (3:1 spleen to myeloma ratio). The mixture was spun down, the rinse was removed, and the pellet was gently resuspended. Polyethylene glycol (PEG 4000) was slowly added and swirled to mix. Saline with glucose was slowly added to the cell suspension. Finally, the cells were spun down and resuspended in hypoxanthine‐aminopterin‐thymidine (HAT) selection medium. The cells were plated in 96‐well plates at 3 × 10^5^ cells/mL.

### 2.3 Screening of hybridomas

After about 2 weeks, supernatant from each well was screened by ELISA for binding to CETN1. Cells from positive wells were then transferred to 24‐well plates, and also plated in soft agar. After 1 week, individual clones were picked from the soft agar and transferred to 96‐well plates. Clones were grown for about 3 days until they were visible by eye then tested by ELISA for binding to CETN1. Positive clones were also tested for binding to CETN2 (Sino Biologicals). Clones which met the criteria of being positive for CETN1 binding and negative for CETN2 binding were expanded and frozen. To test for the presence of CETN1 reactive antibodies 368 hybridoma cultures were screened by ELISA. The capture ELISA was conducted as follows: Costar Corning high binding polystyrene plates (catalogue #9018) were coated overnight with CETN1 antigen, blocked, and probed with supernatant from the hybridomas (the analyte). Finally, binding was detected using mixture of goat/anti‐mouse IgG antibodies (Southern Biotechnology) conjugated to alkaline phosphatase (AP). The antibodies that were used included IgG1, IgG2a, IgG2b, and IgG3. AP labeled goat anti mouse IgM was also used as for detection. The plates were washed between every addition to remove nonspecific binding. Para‐Nitrophenylphosphate (pNPP) was used to indicate the presence of the CETN1‐reactive antibodies.

Ten of the 368 hybridomas screened were found to be positive for the presence of CETN1 antibodies, with some producing IgG, some IgM, and some both. Fifty subclones were then tested for CETN1 and CETN2 binding. 18 of the 50 subclones that produced only IgG were positive for CETN1‐reactive antibodies. These subclones were grown and then frozen at −80°C. A titration was performed with commercial antibodies to CETN1 M01 (Catalogue # H00001068‐M01, Novus Biologicals, Littleton, CO) and M05 (Catalogue # H00001068‐M05, Abnova, Taipei, Taiwan) to determine an optimal concentration of antigen for coating wells in ELISA assays. A concentration of 1.25 µg/mL of CETN1 or CETN2 was determined to be an optimal concentration for the assay. Using these conditions, the binding of the IgMs and IgGs to CETN1 and CETN2 was investigated.

### 2.4 Patients tumor microarray analysis

GeneTex tumor microarray analysis was used to evaluate the binding of the immune serum to PDAC versus normal pancreas. The tissue microarray included 20 cases of PDAC and four sections from normal pancreas in duplicate. Of 20 PDAC cases the anatomical site was pancreas for 19 cases and liver for 1 case. Microwave pre‐treatment (heat‐induced epitope retrieval) was performed for 30 minutes at 90°C on all samples. The serum was diluted 1:1000 and incubated with the microarrays overnight at 4°C. Standard indirect immunoperoxidase procedures were used for immunohistochemistry (ABC‐Elite, Vector Laboratories). Diaminobenzidine was used as a chromogen.

### 2.5 Purification of the CETN1‐specific antibodies and human versus murine CETN1 ELISA

Antibodies 69‐11 and 76‐6 were produced in their respective hybridomas as described above and purified on a protein A column. The binding of 69‐11 antibody to human versus murine CETN1 was evaluated. For this human CETN1 (hRP‐T0488‐EF042 lot 11912K11) was purchased from GeneCopoeia; and the murine CETN1 (854M lot: 218873)—from Creative BioMart. The comparative ELISA was performed using the conditions described above in Screening of hybridomas. Murine IgG MOPC21 was used as a negative control.

### 2.6 Radiolabeling of CETN1‐specific antibodies

Antibodies 69‐11, 76‐6, and the isotype‐matching control murine IgG MOPC21 were conjugated with the bifunctional chelating agent N‐[2‐amino‐3‐(p‐isothiocyanatophenyl)propy1]‐trans‐cyclohexane‐1,2‐diamine‐N,N′,N′′,N′′′,N′′′′‐pentaacetic acid (CHXA") (Macrocyclics, Dallas, TX) for subsequent radiolabeling with ^213^Bi or ^177^Lu. ^225^Ac for construction of the ^213^Bi/^225^Ac radionuclide generator was purchased from Oak Ridge National Laboratory, TN. ^213^Bi was eluted from a ^213^Bi/^225^Ac radionuclide generator with a 0.1 M HI solution. The pH of the solution was adjusted to 6.5 with 5M ammonium acetate buffer prior to the radiolabeling of CHXA’’ conjugated 69‐11 and 76‐6 antibodies. ^177^Lu in form of ^177^Lu chloride was acquired from Radiomedix (TX, USA) and incubated for 60 min at 37°C with CHXA”‐conjugated antibodies to achieve quantitative radiolabeling. The radiolabeled antibodies were used immediately with no need for further purification.

### 2.7 MiaPaCa2 PDAC model

Animal experiments were approved by the University of Saskatchewan's Animal Research Ethics Board and adhered to the Canadian Council on Animal Care guidelines for humane animal use. MiaPaCa2, a human pancreatic carcinoma cell line, was purchased from American Type Culture Collection (ATCC, Manassas, VA) and maintained as directed by ATCC. For the animal therapy experiments, MiaPaCa2 cells were thawed and grown for two weeks in T150 flasks until 80%‐90% confluent at 2 × 10^7^ cells/flask. Cells were removed by trypsinization, pelleted by centrifugation at 1100 RPM for 5 minutes at 4°C, then resuspended in BD matrigel (BD Franklin Lakes, NJ) using chilled pipettes and tubes, to a concentration of 5 × 10^7^ cells/mL. Six‐eight weeks old nu/nu female mice on the BALB/c background (Charles River, Willmington, MA) were anesthetized with isoflurane and injected with 3 × 10^6^ cells subcutaneously into the right flank. 90% of the mice injected with MiaPaCa2 cells developed tumors by day 10 post‐inoculation. Mice with tumors averaging 50‐60 mm^3^ were used for imaging and RIT experiments. The radiolabeled antibodies were administered to mice intraperitoneally. It has been demonstrated both in mouse models and in patients that intraperitoneal administration of the radiolabeled antibodies is equal to intravenous is terms of the delivered antibody dose but is better tolerated.^10^, ^11^

Immunohistochemistry of ex vivo MiaPaCa2 tumors. The MiaPaCa2 tumor‐bearing mice were sacrificed, their tumors were collected, fixed in 10% formalin, and embedded into paraffin. Tumor sections of 4 µm thickness attached to slides were dried in 37°C overnight and baked for one hour at 60°C. Deparaffinization was performed with xylene and graded alcohol in addition to heated EDTA buffer (pH 9). The section was then incubated with 69‐11 or MOPC21 primary antibodies (5 µg/mL) for 1 hour at room temperature. After washing with PBS, the sections were incubated with Dako EnVision + secondary antibody attached to HRP (Cat. No. K4001) for 30 minutes at room temperature. After several washes with PBS, the sections was exposed to DAB chromogen for 10 minutes. The sections were then washed with PBS and processed using hematoxylin.^12^ The sections were dehydrated with ethanol (95% and 100%). A few drops of a mounting medium were then added to the section before covered with a coverslip.

microSPECT/CT imaging of MiaPaCa2 tumor‐bearing mice. microSPECT/CT (micro single photon emission computer tomography/computer tomography) images were collected on a MILabs VECTor^4^ (Netherlands) microSPECT/CT scanner and processed using the comprehensive image analysis software package PMOD (version 3.9, PMOD Technologies, Inc, Switzerland). Two MiaPaCa2 tumor‐bearing mice were injected intraperitoneally with 300 μCi ^177^Lu^_^69‐11 and microSPECT/CT imaging was performed at 1, 24, 48, 72, and 168 hours with the mice in the prone position. SPECT data were collected for 20 minutes using an Extra Ultra High Sensitivity Mouse (XUHS‐M) collimator for 20‐350 keV range using spiral trajectories. All SPECT images were reconstructed using 210 keV (11%) ^177^Lu gamma emissions on a 0.4 mm voxel grid with MILabs reconstruction software.

Radioimmunotherapy of MiaPaCa2 tumor‐bearing mice and safety evaluation. For the pilot experiment, the MiaPaCa2 tumor‐bearing mice were randomized into treatment groups of five animals in each and treated with: 50 µCi ^213^Bi‐ CHXA"‐69‐11 mAb, 50 µCi ^213^Bi‐CHXA"‐76‐6 mAb, 30 µg unlabeled CHXA"‐69‐11 mAb, 30 µg unlabeled CHXA"‐76‐6 mAb, 50 µCi free ^213^Bi, or PBS. The tumors were measured in two dimensions with electronic calipers every 2 days. For the follow‐up comprehensive studies, the MiaPaCa2 tumor‐bearing mice were randomized into groups of five animals and treated with: 100 µCi ^213^Bi‐69‐11, 200 µCi ^213^Bi‐69‐11, 200 µCi ^213^Bi‐IgG control, unlabeled 69‐11, 100 µCi ^177^Lu‐69‐11, 200 µCi ^177^Lu‐69‐11, 200 µCi ^177^Lu‐IgG control, or left untreated. A 5:1 µCi/µg specific activity was used and radiochemical purity was >90% via iTLC. The tumor size was measured every 3 days, and hematologic toxicity was assessed on a weekly basis for white blood cell (WBC), platelet counts (PLT), and red blood cell (RBC). At the completion of the observation period (day 50) the mice were sacrificed and their blood was analyzed for signs of possible hepatic toxicity (aspartate transaminase (AST), and alanine transaminase (ALT)) and renal toxicity (BUN, creatinine). Data were analyzed using GraphPad PRISM (v7.04), mean ± SD values was used to generate the figures. For statistical analysis, t test was used to compare treatment groups, the differences with P values < 0.05 were considered statistically significant.

## 3 RESULTS

**Figure 2:**
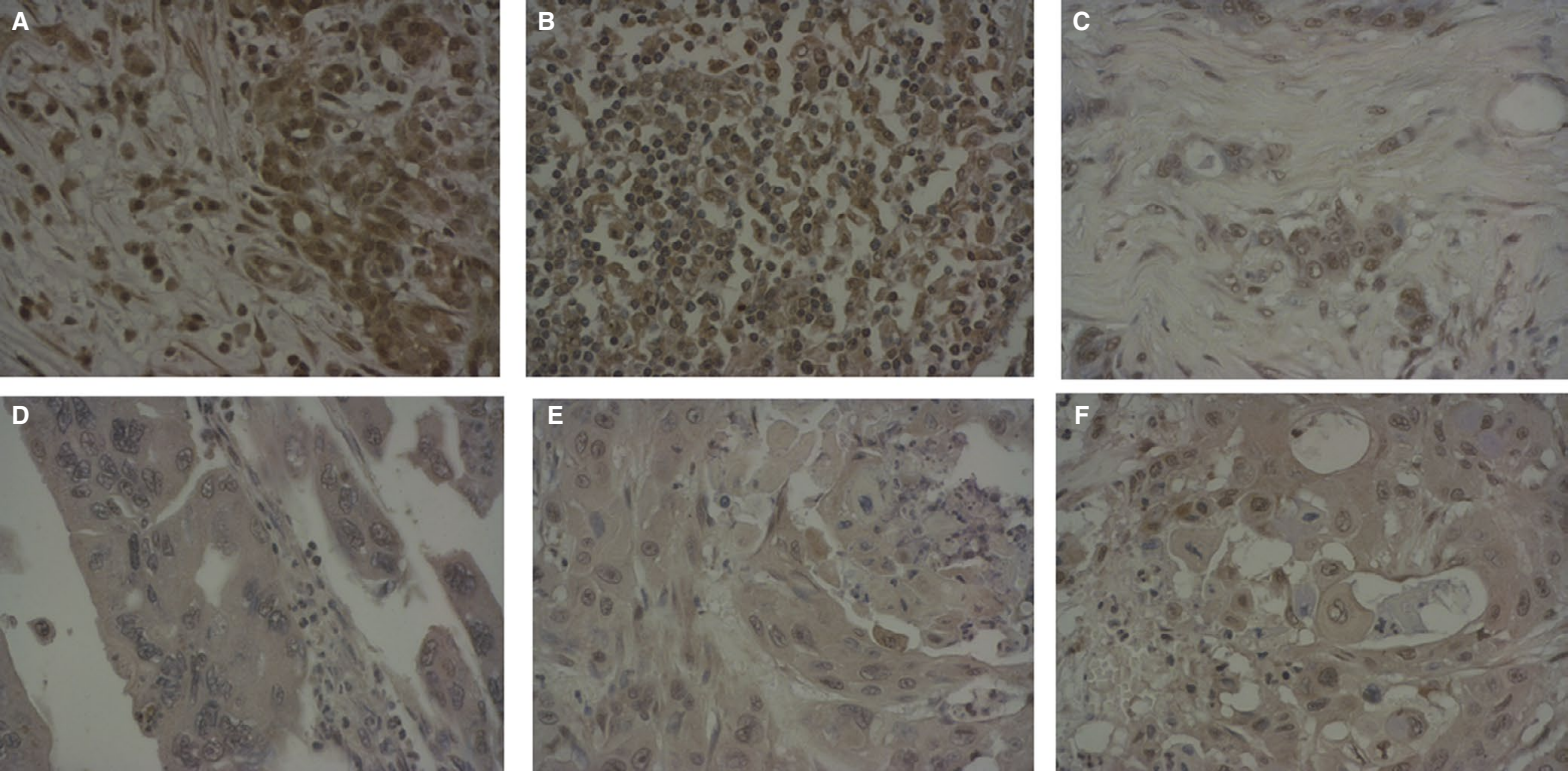
Representative immunohistochemistry images of PDAC tumors and normal pancreas from tumor microarrays stained with immune serum from CETN1‐immunized mice. A and B, Tumors showing positive CETN1 staining; C and D, tumors showing negative CETN1 staining; E and F, normal pancreas showing negative CETN1 staining

Immune serum showed preferential binding to patients PDAC. GeneTex tumor microarray analysis was used to evaluate the binding of the immune serum to PDAC versus normal pancreas. The tumor microarray included total 24 cases in duplicate ‐ PDAC (20 cases) and normal pancreas (four cases). Figure 2A and 2 show examples of tumors with pronounced binding of immune serum while Figure 2C and 2 display tumor with no appreciable binding of the serum. Overall, 10 PDAC cases (50%) demonstrated pronounced binding of immune serum. Importantly, there was no specific binding of immune serum to any of the four cases of normal pancreas (Figure 2E, 2).

Antibodies demonstrated preferential binding to CETN1 versus CETN2. The binding efficiencies of newly generated antibodies to CETN1 versus CETN2 were compared by ELISA. The results for the best CETN1 binding hybridoma clones are shown in Table 1 (IgM isotype antibodies) and Table 2 (IgG isotype antibodies). While the commercially available IgGs M01 and M05 achieved only 1.25‐1.33 CETN1/CETN2 binding ratios (Table 2), several of the newly generated IgM and IgG antibodies had >2 binding ratios, with IgGs 69‐11, 76‐6, and 76‐14 having binding ratios of 7.74, 5.73, and 8.85 respectively.

**Table 1:** Binding of IgM antibodies to CETN1 and CETN2

Clones	CETN1/CETN2 binding ratio
117‐32	2.45
117‐33	2.14
117‐34	2.47
117‐35	2.27
HAT	1.18

**Table 2:** Binding of IgG antibodies to CETN1 and CETN2

Clones	CETN1/CETN2 binding ratio
69‐11	7.74
69‐18	2.82
76‐1	3.72
76‐6	5.73
76‐13	2.49
76‐14	8.85
76‐15	3.91
M01	1.25
M05	1.33

**Figure 3:**
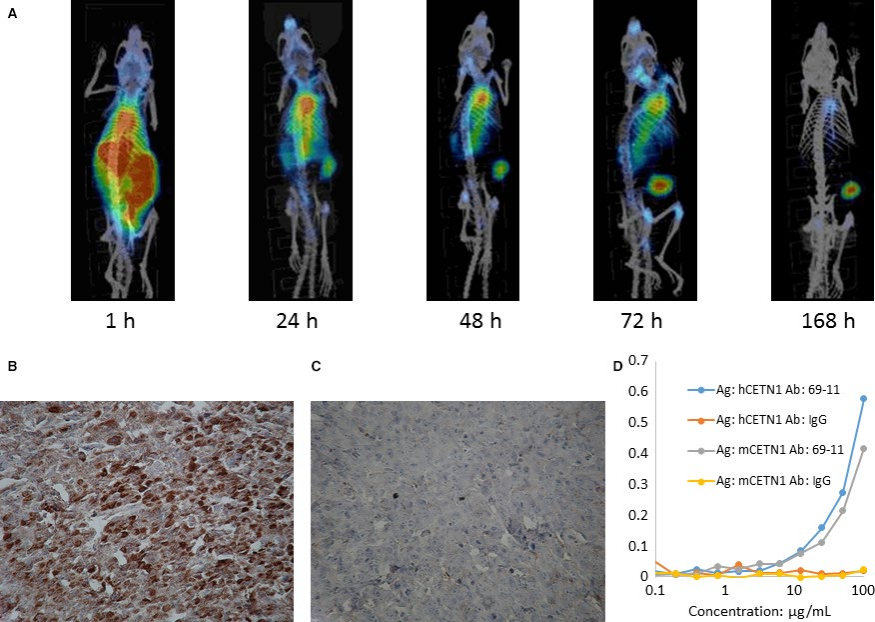
Visualizing CETN1 expression in MiaPaCa2 tumors in vivo and ex vivo: A, microSPECT/CT imaging of MiaPaCa2 tumor‐bearing mouse at 1, 24, 48, 72, and 168 hours post administration of ^177^Lu‐69‐11 antibody to CETN1; B, immunohistochemistry of the MiaPaCa2 tumor stained with 69‐11 mAb; C, the same tumor stained with isotype matching control MOPC21; D, comparative ELISA of 69‐11 mAb binding to human and murine CETN1. Murine IgG MOPC21 was used as a negative control

Antibody 69‐11 localized to PDAC xenografts in mice in vivo and ex vivo. To investigate if the newly generated antibodies could localize in PDAC xenografts in mice, nude mice bearing MiaPaCa2 PDAC xenografts were injected with 300 μCi ^177^Lu‐labeled 69‐11 antibody and imaged by microSPECT/CT at 1, 24, 48, 72, and 168 hours (Figure 3A). Although most of the ^177^Lu‐69‐11 antibody was still in circulation at 1 hour post administration, it clearly localized to the tumors in the right flank of the mice by 24 hours, with the tumor uptake progressively increasing until 72 hours. Pronounced tumor uptake was still detectable at 168 hours (7 days) post injection of the antibody. This imaging study provided impetus for performing RIT of PDAC xenografts with radiolabeled antibodies to CETN1. Furthermore, the ability of 69‐11 to bind CETN1 specifically was confirmed by immunohistochemistry on ex vivo MiaPaCa2 tumors. Figure 3B displays the staining of CETN1 with 69‐11 in the tumor, and its absence – when isotype control mAb MOPC21 was used (Figure 3C). The comparative ELISA of 69‐11 mAb binding to human and murine CETN1 demonstrated that 69‐11 mAb bound to both human and murine CETN1 (Figure 3D).

**Figure 4:**
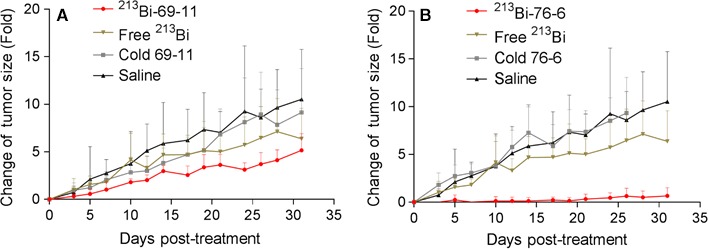
Pilot radioimmunotherapy study of MiaPaCa2 tumor‐bearing mice with ^213^Bi‐labeled antibodies to CETN1. Relative tumor size change (Fold) when treated with ^213^Bi‐labeled antibodies, free ^213^Bi, cold antibodies, and Saline. A, treatment groups with 69‐11 antibody; B, treatment groups with 76‐6 antibody

Radioimmunotherapy of PDAC xenografts was effective, safe, and CETN1‐specific. Initially we performed a pilot RIT experiment with IgG antibodies 69‐11 and 76‐6, which possessed high binding specificity to CETN1 over CETN2. MiaPaCa2 tumor‐bearing mice with tumor volumes of approximately 50 mm^3^ were treated intraperitoneally (IP) with 50 μCi of alpha‐emitter ^213^Bi‐labeled 69‐11 or 76‐6 antibodies, along with unlabeled antibodies, free ^213^Bi, and saline for controls. Figure 4 shows the changes in the tumor volume for ^213^Bi‐69‐11 (Figure 4A) and ^213^Bi‐76‐6 (Figure 4B), compared to the control groups. It is obvious that in spite of the low radioactive payload of 50 μCi, both radiolabeled antibodies had a profound effects on tumor growth rate, which was significantly (*P* < 0.0005) reduced in comparison to the untreated controls and unlabeled antibody. Free ^213^Bi did not have any significant effect on tumor retardation (*P* = 0.16) pointing to the importance of the targeted delivery of radioactivity to the cells by the antibodies.

**Figure 5:**
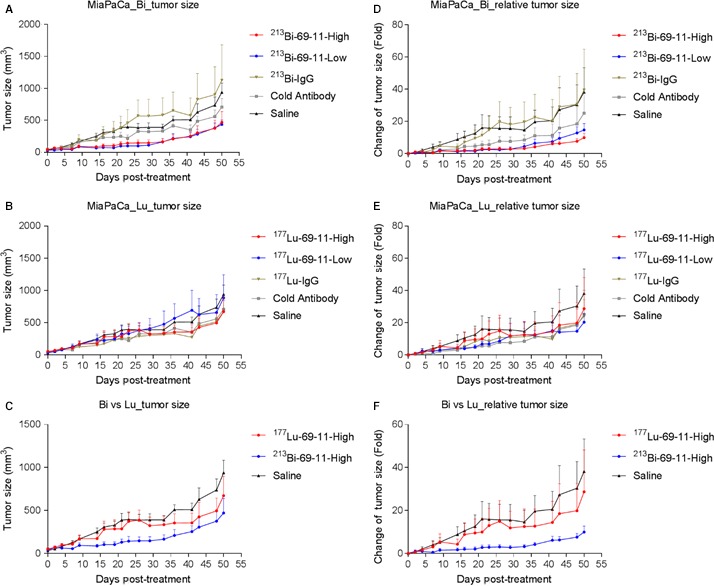
Radioimmunotherapy of MiaPaCa2 tumor‐bearing mice with ^213^Bi‐ and ^177^Lu‐labeled 69‐11 antibody to CETN1. A and D, Tumor size for groups treated with ^213^Bi‐69‐11, ^213^Bi‐IgG, cold 69‐11 antibody, and saline; B and E, tumor size for groups treated with ^177^Lu‐69‐11, ^177^Lu‐IgG, cold 69‐11 antibody, and saline; C and F, comparison of tumor size between ^213^Bi‐69‐11 and ^177^Lu‐69‐11 treatment groups. High: 200 µCi, Low: 100 µCi

**Figure 6:**
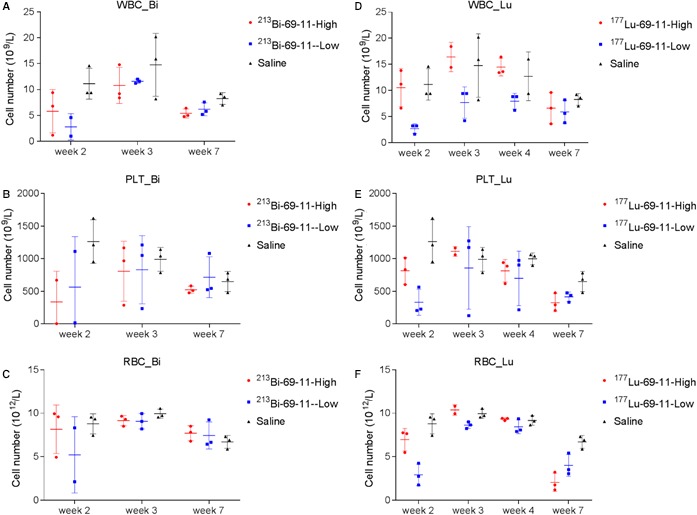
Blood chemistry analyses during radioimmunotherapy of MiaPaCa2 tumor‐bearing mice with ^213^Bi‐ and ^177^Lu‐labeled 69‐11 antibody to CETN1. A, WBC, ^213^Bi‐69‐11; B, platelet, ^213^Bi‐69‐11; C, RBC, ^213^Bi‐69‐11; D, WBC, ^177^Lu‐69‐11; E, platelet, ^177^Lu‐69‐11; F, RBC, ^177^Lu‐69‐11

Following the encouraging results of the pilot experiment, a more comprehensive RIT experiment was conducted. Clone 69‐11 was chosen for the follow‐up experiment because of the higher productivity of the 69‐11 hybridoma. The goal of the experiment was to compare the efficacy and safety of the 69‐11 antibody when radiolabeled with two different radionuclides—a short lived alpha emitter ^213^Bi (46 mintes half‐life), and a long‐lived beta emitter ^177^Lu (6.7 days half‐life). Labeling with ^213^Bi converted 69‐11 antibody into a very effective RIT reagent with tumor growth rate significantly (*P* = 0.0001) reduced by a single injection of either 100 or 200 μCi (Figure 5A,D). Importantly, the effect of ^213^Bi‐69‐11 on the tumor was CETN1‐specific, as 200 μCi control IgG had no effect on the tumor growth (*P* = 0.96). In spite of impressive localization of ^177^Lu‐69‐11 in the tumor, as demonstrated during the imaging experiments (Figure 3A), it was not very effective in slowing down the tumor growth, which shows no difference between ^177^Lu‐69‐11 and ^177^Lu‐IgG control (*P* = 0.54) (Figure 5B,E). Furthermore, ^177^Lu‐69‐11 was several fold less effective than ^213^Bi‐69‐11 (Figure 5C,F). Both ^213^Bi and ^177^Lu groups showed only transient hematologic toxicity (Figure 6) and absence of liver and kidney toxicity (Figure S1) attesting to the very high safety margin of targeting CETN1 with RIT.

## 4 DISCUSSION

The discovery of *CETN1* mRNA level being upregulated 25‐fold in PDAC tumors compared to normal pancreas 2 opened new opportunities for diagnosis and therapy of PDAC by targeting CETN1. However, the lack of specific antibodies to CETN1 hindered the development of this new therapy. Our newly developed antibodies to CETN1 with minimal cross‐reactivity to CETN2 allowed us to demonstrate for the first time, using tumor microarray analysis which translate the qPCR data into the CETN1 protein expression, by 50% of PDAC tumors, in contrast to non‐expression of CETN1 by normal pancreas. The availability of these novel CETN1‐specific antibodies enabled us to perform the pilot evaluation of these antibodies for radioimmunoimaging and RIT of experimental PDAC.

Multiple clinical trials of targeted radionuclide therapy of pancreatic cancer including RIT and peptide receptor radionuclide therapy (PRRT) with antibodies and peptides as radiation targeting vehicles, respectively, have been performed in the last decade, and have demonstrated the safety and potential efficacy of targeted radionuclide therapy for treatment of this formidable disease (reviewed in Ref. 13, 14, 15, 16). Considerable progress has been made in the treatment of pancreatic neuroendocrine tumors with radiolabeled somatostatin analogues entering the realm of standard clinical care in the US and Europe with the approval of Lutathera, However, PDAC remains a major challenge. The RIT clinical trials for treatment of PDAC, though demonstrating the safety of the RIT approach in PDAC patients, have thus far produced lackluster therapeutic outcomes. The reasons for this are multiple, including the overall aggressive nature of the disease, as well as poor vascularization and other challenging aspects of the PDAC stroma and microenvironment.^17^ In addition; the historical choices for antigen targets, and the physical properties of the radionuclides selected for delivery of the cytotoxic radiation, might have also contributed to the modest therapeutic outcomes, thus far, of RIT in PDAC. In this regard, widely utilized RIT target antigens such as anti‐carcinoembryonic antigen 18 or mucin glycoprotein 19 are also expressed in multiple normal tissues, which potentially causing difficulty in escalating treatment doses without concurrent off target toxicity issues. The beta‐emitting radionuclides historically chosen for radiolabeling of targeting antibodies ‐ ^131^I, ^90^Y, and ^177^Lu, with their long range energy deposition characteristics and relatively long half‐lives that result in a prolonged duration of radiation delivery, might not be physically and radiobiologically effective enough to deliver adequately cytotoxic radiation doses to counteract the aggressive nature of PDAC.

The concept of targeting intracellular antigens with the radiolabeled mAbs was introduced by Alan Epstein in 1989.^20^ It is based on the notion, that in rapidly growing tumors high rates of cellular turnover and cell necrosis release intracellular antigens into the extracellular space, where they are accessible to radiolabeled mAbs to these antigens. Such antibodies deliver cytotoxic radiation to nearby malignant cells via the so called “cross‐fire” effect. Furthermore, this strategy is attractive because intracellular antigens in normal tissues with very little cellular turnover are not accessible to mAbs by virtue of their “safe” intracellular location. In our laboratory we have successfully targeted several intracellular antigens with radiolabeled antibodies for the purposes of RIT: melanin in metastatic melanoma both experimentally and in Phase I clinical trial,^21^, ^22^, ^23^ E6 and E7 intranuclear viral proteins in human papilloma virus (HPV)‐positive cervical and head and neck cancers,^24^, ^25^ and single strand DNA in experimental PDAC.^26^ Additional advantage of targeting intracellular antigens is that after each administration of RIT there are more dead or dying cells in the tumor which can be targeted with the next RIT dose, thus making such treatment “target‐generating” in contrast with “target depleting” when surface antigens are targeted. In our study, we targeted CETN1, which by virtue of being a Cancer/Testis Antigen (CTA), is not expressed in healthy tissues other than testes. This feature should facilitate the administration of highly effective doses of radiolabeled antibodies, without limiting adverse effects from off target, normal tissue toxicity. Additionally, effective tumor responses from CETN1 targeted radiation might not even require the administration of high radionuclide doses, since the radiolabeled antibody will not be “wasted” by targeting the antigen on the healthy tissue rather than concentrate in the tumor. The lack of hematological and systemic toxicity during treatment of MiaPaCa2 tumor‐bearing mice with ^213^Bi‐labeled 69‐11 antibodies in our current study provides proof of this concept (Figure 6 and Figure S1).

In the last decade the use of alpha‐emitting radionuclides has been gaining momentum, both in clinical trials and in preclinical studies. This development is driven by appreciation of the advantages of alpha‐emitters over beta‐emitters, including very precise targeting of the cancer cells due to the alpha‐particles’ short 50‐80 µm tissue range, and increased killing efficiency due to high linear energy transfer (reviewed in Ref. 27, 28, 29). The first pre‐clinical work on using an alpha‐emitter ^212^Bi for treatment of experimental pancreatic cancer was described by Kurtzman et al^30^ 30 years ago, and recently there has been a resurgent interest towards using alpha‐emitters for targeted radionuclide therapy of PDAC.^26^, ^31^, ^32^

In our study, we performed side by side comparison of the short‐lived alpha‐emitter ^213^Bi (half‐life 46 minutes), and the long‐lived beta‐emitter ^177^Lu (half‐life 6.7 days). Our results indicate that ^213^Bi is a much more effective radionuclide for CETN1 targeted alpha therapy of PDAC. A possible explanation for this striking difference in efficacy could be the ability of the short lived ^213^Bi nuclide to deliver its radiation dose in a short period of time; thereby allowing its intense, high relative biological effectiveness (RBE) to counteract the aggressive growth of PDAC. By contrast, we surmise that, in our study, the aggressive PDAC tumor growth could not be effectively controlled by the relatively protracted mode of delivering lower RBE beta radiation generated by ^177^Lu.

Interestingly, in our earlier studies we used the same MiaPaCa2 xenografted model to test single strand DNA antibody, radiolabeled with ^213^Bi, as a possible agent for RIT of PDAC.^26^ Though this molecular targeted radiation treatment agent was also effective; several‐fold more ^213^Bi activity was required to achieve the same tumor suppress effect comparable to what we observed in the current study. This observation illustrates the superior nature of CETN1 as the target, which facilitate the majority of the radioimmunoconjugate concentrating in the PDAC tumor site; due to the high specificity of the antibody as well as the exclusive accessibility of CETN1 antigen within the PDAC microenvironment.

In conclusion, we report the generation of novel highly specific antibodies to CETN1, their evaluation in patients’ tumor microarrays, their utility in radioimmunoimaging, and effectiveness in RIT of experimental PDAC. The results demonstrate the ability of these antibodies to detect CETN1 in vitro and in vivo; as well as their use in the highly efficacious and safe treatment of experimental PDAC when radiolabeled with an alpha‐emitter, ^213^Bi. Further evaluation of these novel reagents for their potential in diagnosis and treatment of PDAC is warranted.

## CONFLICT OF INTEREST

DR, RAB and ED are co‐inventors on the US patent application “Antibodies to Cetn1, methods of making and uses thereof ”

## AUTHOR CONTRIBUTIONS

DR and ED came up with the concept of the study; RJ, KJHA, MEM, MH, ZJ, KS, SVB and RAB performed experiments; RAB and ED analyzed the data; ED and DR wrote the manuscript.

## Data Availability

The data that support the findings of this study are available from the corresponding author upon reasonable request.
