# Supplementary figures and images for: Evaluation of novel highly specific antibodies to cancer testis antigen Centrin‐1 for radioimmunoimaging and radioimmunotherapy of pancreatic cancer
Source: Cancer Med. 2019 Jul 16;8(11):5289–300. doi: 10.1002/cam4.2379 (PMC6718527; doi:10.1002/cam4.2379)

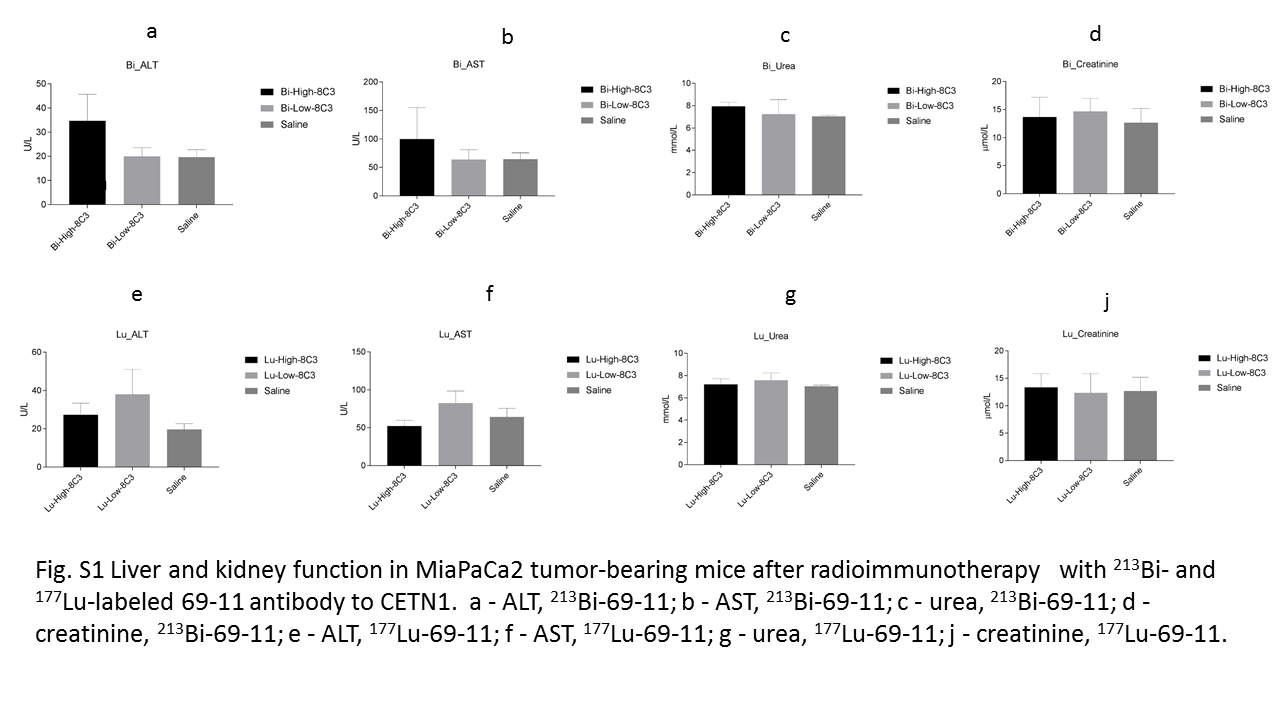

Supplement: Supplementary file 1 [file CAM4-8-5289-s001.tif]
